# A Critical Appraisal on the Role of Varicocele in Male Infertility

**DOI:** 10.1155/2012/597495

**Published:** 2011-11-28

**Authors:** Ricardo Miyaoka, Sandro C. Esteves

**Affiliations:** ANDROFERT—Center for Male Reproduction, Av. Dr. Heitor Penteado 1464, Campinas 13075-460, São Paulo, Brazil

## Abstract

Varicocele is a major cause of male infertility, as it may impair spermatogenesis through several distinct physiopathological mechanisms. With the recent advances in biomolecular techniques and the development of novel sperm functional tests, it has been possible to better understand the mechanisms involved in testicular damage provoked by varicocele and, therefore, propose optimized ways to prevent and/or reverse them. Up to now, there is still controversy involving the true benefit of varicocele repair in subfertile men as well as in certain specific situations such as concomitant contralateral subclinical varicocele or associated nonobstructive azoospermia. Also, with the continued development of assisted reproductive technology new issues and questions are emerging regarding the role of varicocelectomy in this context. This paper reviews the most recent data available on the pathogenesis, diagnosis, and management of varicocele with regard to male infertility.

## 1. Introduction

Approximately 8% of men in reproductive age seek medical assistance for fertility-related problems. Among them, 1%–10% carry a condition that compromise their fertility potential and varicocele alone accounts for 35% of these cases [1, 2]. Our personal database of a referral tertiary center for male reproduction presents an incidence of 21.9% of varicocele in 2,875 analyzed subjects [3].

While varicocele has an incidence of 4.4%–22.6% in the general population, 21%–41% of men with primary infertility and 75%–81% of those with secondary infertility have this condition [4, 5]. 

The impact of varicocele on male fertility was not suspected until the end of the 19th century, when occlusion of dilated veins from the pampiniform plexus was shown to improve semen quality [4, 6]. MacLeod in 1965 demonstrated decreased sperm count, decreased motility and higher prevalence of abnormal forms in semen specimens collected from infertile men with varicocele [7]. 

Although the physiopathology of varicocele and its relationship with male infertility has been discussed for the last 50 years, the exact mechanisms that would ultimately lead to an infertile/subfertile state are still controversial. Even more debatable is the true benefit from its surgical repair [8, 9]. Treating male factor infertility should have as its ultimate goal to achieve a live birth. However, efforts must be made to maximize the couple's fertility potential. In this sense, it is argued that varicocele treatment may be critical to restore or optimize testicular function.

We present a review on the current concepts and controversies surrounding varicocele as a condition affecting male fertility.

## 2. Epidemiology: An Overview

Varicocele is identified in 7% and 10%–25% of prepubertal and postpubertal males, respectively [10, 11]. The higher frequency in elderly males and in men with secondary infertility suggests that it is a progressive disease [12, 13].

Anecdotal experience suggesting that lean men are more prone to varicocele has been supported by recent studies showing that varicocele occurrence is inversely correlated with body mass index [14, 15]. A higher prevalence in first-degree relatives has also suggested an inherited pattern [13]. 

Also, it has been shown that long-term intense physical activity (2–4 hours daily, 4-5 times a week, during 4 years) worsened semen quality in men with varicocele [16].

## 3. Physiopathology: Novel Concepts

Despite the several different theories that aim to explain the impact of varicocele on testicular function, none can fully clarify the variable effect of varicocele on human spermatogenesis and male fertility. Proposed mechanisms include hypoxia and stasis, testicular venous hypertension, autoimmunity, elevated testicular temperature, reflux of adrenal catecholamines, and increased oxidative stress [17].

Venous hypertension, defined as the hydrostatic column that entails pressure over the already sick gonadal venous valves, along with reflux of toxic adrenal and renal metabolites into the testicles, can cause chronic vasoconstriction of testicular arterioles [18]. This phenomenon leads to persistent hypoperfusion, stasis and hypoxia, and subsequent dysfunction of the spermatic epithelium [17, 19]. Elevated testicular temperature in men with reduced sperm quality and varicocele have been demonstrated as well as the reduction in temperature following varicocele repair [20]. However, the mechanism by which temperature affects spermatogenesis is not clearly understood.

Koksal et al. biopsied varicocele-affected testicles and showed a decrease in E-cadherin and alpha-catenin in the Sertoli-Sertoli cell junction as well as disruption of the blood-testis barrier [21]. Musalam et al. reported an ultrastructural comparative analysis of morphological changes in normal internal spermatic veins and grade 3 varicocele veins assessed by scanning and transmission electron microscopy. Varicocele veins showed narrowing/obliteration of their lumens, destruction of the endothelial cells, invagination of the intima, and deposition of collagen bundles in the media layer. Ultrastructural changes included elongation of the endothelial cells with features of cellular damage, loss of the internal elastic lamina, and the appearance of ghost bodies and degenerative vacuoles in the subendothelial layer [22].

Excessive oxidative stress is commonly seen in infertile men with varicocele. High production of reactive oxygen species (ROS) and a decrease in total antioxidant capacity (TAC) impairs the fluidity of the sperm plasma membrane and the integrity of deoxyribonucleic acid (DNA) in the sperm nucleus. Fertility markers assessed *in vitro*, such as fertilization rate, embryo cleavage, implantation, pregnancy, and live birth rates are all negatively affected by abnormal high levels of sperm DNA damage [23].

An imbalance between ROS production and TAC decrease has also been implicated as the result of acidification of spermatozoa cytosol and seminal plasma in men with varicocele [24]. Oxidative stress via ROS, especially lipid peroxidation, damages membrane function in sperm head and midpiece altering morphology and impairing motility, but also leads to a decrease in intracellular pH. The ideal pH for ROS scavenging activity by the enzymatic antioxidant systems ranges from neutral to slightly alkaline, being markedly depressed in acidic states. Impairment of TAC may reflect as further decrease in sperm motility [23]. These effects, however, have been speculated to vary from one subject to another according to their capacity to counteract the deleterious effects of membrane dysfunction and DNA damage. This may help understand the variable effect of varicocele on male infertility.

Recent findings reported by Blumer et al. confirmed previous reports of a negative correlation between sperm morphology and the percentage of sperm with high DNA fragmentation (*r* = −0.450) in men with varicocele [25]. An increase in oxidative stress determined by the rise in malondialdehyde, the major product of lipid peroxidation, was not observed in the aforementioned study although a decrease in mitochondrial activity and acrosome integrity was documented. Smith et al. found that high levels of sperm DNA damage were associated with varicocele even when semen analysis had been normal [26]. Semen analysis results, as routinely performed, are limited in its validity as surrogate for the assessment of male fertility potential. For this reason, it has been suggested that sperm function tests, such as sperm DNA integrity, are better indicators of male fertility potential and should be, therefore, included in the semen evaluation [27, 28].

In a study involving men with palpable varicocele and oligozoospermia, Smit et al. showed significant improvement in DNA fragmentation index (DFI) 3 months after varicocelectomy (preoperative %DFI: 35.2 ± 13.1 versus postoperative %DFI 30.2 ± 14.7, *P* = 0.019) [29]. A difference could also be noted between couples who conceived spontaneously or with assisted reproductive technology (ART) compared to those who failed (DFI%: 26.6% ± 13.7 versus 37.3% ± 13.9, *P* = 0.013). However, these authors demonstrated that not all infertile patients had a decrease in sperm DNA damage after varicocele repair. In a recent work by Dada et al. studying 11 men with clinical varicocele, surgical repair resulted in rapid (1 month) significant decline in free-radical levels followed by slow (3–6 months) significant decline in DNA damage assessed by Comet assay [30]. On the basis of their findings, the authors of the aforementioned study recommended that infertile couples whose male partner had varicocele repair should wait 6 months after surgery before attempting to conceive. 

Sperm DNA fragmentation could also result from aberrant chromatin packaging during spermatogenesis or be a consequence of the triggering of an apoptotic-like process by ROS overproduction. Sadek et al. assessed the rate of chromatin condensation using aniline-blue staining in infertile men with varicocele and showed significant improvement following surgical correction of grade III varicose veins [31].

## 4. Varicocele and Infertility: A Controversial Issue

The idea supporting varicocele relationship with infertility stands on three aspects:

1. the increased incidence of varicocele among infertile men,
2. the association between varicocele and reduced semen parameters and testicular size,
3. the improvement of semen parameters and pregnancy rates following surgical correction of clinical varicocele.

A varicocele incidence of 25.6% was observed in a large observational study involving more than 9,000 men [32]. In this study, men with varicocele were also shown to have lower total sperm count and testosterone levels, as well as reduced testicular size on the same side of varicose vessels compared with those without varicocele. 

The hypothesis that varicocele can cause testicular damage was further confirmed on pubertal boys in which the reduction in testicular size ipsilateral to the pathology was restored after surgical repair [33]. It should be noted, however, that catch-up testicular growth among adolescents following varicocele repair is not universal and may be dependent on several factors including patient age [34]. Discrepant testicular size in adolescents with varicoceles firstly has to be documented as worsening or persisting in relation to ipsilateral varicoceles. Accordingly, some investigators have recommended to follow-up postoperative serum androgens to better assess testicular function after varicocele repair [34].

In a study involving 30 men with a left-sided grade 3 varicocele, conventional and nonconventional seminal parameters were compared before and after subinguinal microsurgical varicocelectomy with 30 normozoospermic presumptively fertile match controls without varicocele [35]. Four months after surgical repair, patients showed higher sperm density, total sperm count, percentage of normally shaped spermatozoa and spermatozoa with progressive motility compared to baseline. Interestingly, all conventional semen parameters examined remained significantly lower after surgery compared to controls. Biofunctional parameters that are not routinely evaluated also improved after varicocele repair. Lower proportions of spermatozoa with early signs of apoptosis, assessed by mitochondrial membrane potential and phosphatidylserine externalization, were observed in the varicocele group after surgical repair. Moreover, postoperative results revealed increased proportion of spermatozoa with condensed chromatin and nonfragmented DNA as compared to baseline in men with treated varicocele [35]. 

Cho et al. analyzed the effects of microsurgical varicocelectomy for causes other than infertility in 121 men with clinical varicocele and at least one abnormal semen parameter [36]. Overall, seminal improvement was observed in 76% of subjects following surgery. Subgroup analysis of patients with oligozoospermia, asthenozoospermia, or teratozoospermia also showed significant improvement in semen parameters after varicocele repair. 

In a well-designed prospective randomized controlled trial by Abdel-Meguid et al., 145 participants underwent varicocele treatment versus observation [37]. Microscopic subinguinal technique was used, and only men with female partners under 35 years of age were included. These authors showed that spontaneous pregnancy was achieved in 13.9% of the control arm compared with 32.9% of the treatment arm, with an odds ratio of 3.04 (95% CI, 1.33–6.95) in favor of varicocele treatment. Improvement in semen parameters was also detected, but the authors emphasized pregnancy as being the ultimate goal for infertility patients with treated varicoceles as semen parameters demonstrated extensive intra- and interindividual variability as well as overlapping between fertile and infertile men.

Despite the above mentioned, it remains unclear the reasons why about 2/3 of men with varicocele retain their fertility [5, 9] and not all of them achieve fertility improvement after varicocelectomy [38, 39]. Moreover, reports on the ineffectiveness of varicocele treatment to increase the chances of conceiving are intriguing. In a meta-analysis by Evers and Collins [40], no benefit from varicocele treatment was verified regarding odds of pregnancy. However, a major critique to this review was the inclusion of patients with subclinical varicocele and/or normal semen characteristics [41].

## 5. Diagnosis: Where Do We Stand?

Physical examination with the patient standing in a warm room is currently the preferred method for varicocele diagnosis and has a sensitivity and specificity of around 70% compared with other diagnostic tools [42, 43]. The term clinical varicocele refers to those detectable by either visual inspection or palpation. The most widely used classification is the Dubin grading system [44]:

Grade 3: visible and palpable at rest,
Grade 2: palpable at rest,
Grade 1: palpable during Valsalva maneuver,
Subclinical: not palpable or visible at rest or under Valsalva maneuver but detectable by Doppler ultrasound.

Whenever physical examination is inconclusive or difficult to perform as in cases of low-grade varicocele, previous scrotal surgery, obesity, concomitant hydrocele, or scrotal tenderness/hypersensitivity, imaging studies are recommended. Among the noninvasive modalities, color Doppler ultrasound (CDU) has been shown to be the best diagnostic tool. Using a cutoff value of 3 mm for vein diameter, CDU has a sensitivity of about 50% and specificity of 90% compared to physical examination [45]. Pilatz et al. using a 7 MHz transducer determined an optimal cutoff point for discriminating testicles with or without clinical varicocele in the relaxed supine position of 2.45 mm (sensitivity 84%, specificity 81%) and 2.95 mm during Valsalva maneuver (sensitivity 84%, specificity 84%) [46]. A pencil probe Doppler (9 MHz) is an inexpensive tool that may be useful in helping diagnosing varicocele. Examination should be carried out with the patient standing and a venous “rush” produced by blood reflux should be heard under Valsalva maneuver. Although simple, this method was also shown to be positive in men harboring subclinical varicocele [47]. We advocate the use of pencil probe Doppler to assess subclinical varicocele on the contralateral side in a patient who already has a clinical dilation in order to decide for bilateral surgical repair or not [48]. However, the clinical significance of a positive result for venous reflux as shown by adjuvant diagnostic modalities in infertile men is uncertain.

## 6. Treatment: Options and Outcomes

Treatment of varicocele in infertile men aims to restore or improve testicular function. Current recommendations propose treatment for couples with documented infertility whose male partner has a clinical varicocele and at least one abnormal semen parameter. Men who are not attempting to achieve conception but fit into this description and have a desire for future fertility are also candidates for varicocele repair [49–51].

Nonsurgical treatment modalities for varicocele-related infertility are poorly studied, and there is a need for well-designed trials. L-carnitine in combination with the nonsteroidal anti-inflammatory agent cinnoxicam have been studied but failed to show improvement in seminal parameters in men with clinical varicocele [52]. Use of oral kallikrein (600 units per day) for 3 months improved both sperm motility and morphology in a group with left-sided varicocele and asthenozoospermia [53]. Early use of menotropin in combination with surgical repair provided additional improvement in semen parameters when compared with varicocelectomy alone [54]. More recently, the use of vitamins and antioxidant agents have been used to treat infertile men with varicocele. Daily oral administration of pentoxifylline, zinc, and folic acid for 3 months improved sperm morphology [55]. In another work by Paradiso Galatioto et al., a combination of vitamins and minerals was able to improve sperm count in men with persistent oligozoospermia following varicocele embolization but did not increase pregnancy rates at 1-year followup [56].

The gold standard treatment currently accepted for varicocele is surgical repair either by open approach associated or not with magnification, laparoscopy, or through percutaneous embolization of the internal spermatic vein. Regardless of the chosen technique, the ultimate goal relies on the occlusion of the dilated veins that compose the pampiniform plexus. The high retroperitoneal (Palomo), radiologic, and laparoscopic approaches allow the ligation of the gonadal vein. The inguinal (Ivanissevich) and subinguinal approaches permit ligation of the external spermatic and cremasteric veins that may contribute to the varicocele and may play a role in recurrence. 

Percutaneous embolization offers a rapid recovery and can be successfully accomplished in approximately 90% of attempts. However, the technique demands interventional radiologic expertise and has potential serious complications, including vascular perforation, coil migration, and thrombosis of pampiniform plexus [57–59].

Laparoscopic varicocelectomy provides high magnification, but hydrocele formation and recurrence can occur in up to 10% and 5% of cases, respectively. Also, specific training and high-cost materials are needed. Finally, although considered a minimally invasive approach for several abdominal pathologies, it is considerably more invasive than an open microsurgical approach and is related to the inherent complications of a laparoscopic procedure such as vascular and intestinal injuries [59, 60].

Open surgical varicocelectomy is performed by retroperitoneal, inguinal, or subinguinal approaches. The retroperitoneal high ligation of the internal spermatic vein (Palomo's technique) although easy to perform is associated with high recurrence and hydrocele formation rates. Inguinal and subinguinal approaches allow for ligation of external spermatic vessels. The addition of magnification to both techniques permits better visualization and sparring of internal spermatic arteries and lymphatics, thus avoiding testicular atrophy and hydrocele formation [61]. The main difference between the subinguinal and the inguinal approach is that the opening of the external oblique muscle aponeurosis is avoided in the former, which may implicate in shorter and less painful postoperative recovery. However, there is no objective data to substantiate a clear advantage of one technique over the other. 

A systematic review involving 4,473 men aimed to define the best treatment modality of palpable varicocele in infertile men [59]. The authors concluded that open inguinal or subinguinal artery and lymphatic sparing microsurgical techniques resulted in higher spontaneous pregnancy rate, fewer recurrence and complications compared to laparoscopic, radiologic embolization, macroscopic inguinal, or retroperitoneal procedures. Similarly, a recent comparative review among surgical techniques for varicocele repair involving 33 studies and over 5,000 patients concluded that by using either of the two microsurgical techniques, inguinal or subinguinal, better outcomes across all parameters postoperatively were obtained including higher pregnancy rates (44.75%; range 33.8%–51.5%) and lower recurrence and hydrocele formation rates (2.07% and 0.72%, resp.) compared to other treatment modalities [62].

Varicocelectomy is believed to improve one or more semen parameters in 65% of those men who are treated [63]. The mean time for semen improvement and spontaneous pregnancy after surgery is approximately 5 and 7 months, respectively [64]. The reasons why fertility potential is not always improved are still obscure, and consistent data is lacking to determine prognostic factors that might help identify the best candidates for treatment. It seems that infertile men either with higher preoperative semen parameters or undergoing surgery for large varicocele are more likely to benefit from varicocelectomy [63–65]. Men who achieve a total motile sperm count greater than 20 million were more likely to conceive spontaneously or through intrauterine insemination after varicocelectomy [65]. An interesting report suggested that advanced paternal age does not influence outcomes of men with varicocele-associated infertility. It should be noted, however, that results might be biased by the fact that the group of men older than 40 years had a significantly higher proportion of men with secondary infertility as opposed to the other with younger individuals [66]. Correction of both sides at the same operative time has also been advocated when bilateral palpable varicocele is encountered [67].

Assisted reproductive technology (ART), including *in vitro* fertilization (IVF) and intracytoplasmic sperm injection (ICSI), is routinely used to treat male factor infertility. Because of the success of ART, the optimal method to achieve pregnancy with male infertility has been debated. Decision analysis-based comparisons of ART and varicocelectomy suggest that varicocele repair is more cost-effective than the use of ART in men with impaired semen parameters [68, 69], which should be taken into consideration in patient care. In addition, the indication of varicocele repair prior to IVF/ICSI may be considered in certain circumstances. Men with nonobstructive azoospermia (NOA) and favorable testicular histopathology may resume sperm production following repair of clinical varicocele [70]. Sperm restoration, even if minimal, yields the possibility of IVF/ICSI without the need of sperm retrieval techniques (SRT). It has been shown that for patients who are still azoospermic after varicocelectomy, SRT success rates using testicular microdissection sperm extraction, and as a result, the couple's chance for pregnancy may be increased [71]. Varicocelectomy has also a potential to obviate the need for ART or to downstage the level of ART needed to bypass male factor infertility [72]. Recently, it has been shown that treatment of clinical varicocele may also improve the outcomes of assisted reproduction in couples with varicocele-related infertility. Esteves et al. studied 242 infertile men with treated and untreated clinical varicocele who underwent intracytoplasmic sperm injection (ICSI) and found significantly higher live birth rates after ICSI in the group of men who underwent artery and lymphatic sparing subinguinal microsurgical varicocele repair before ART (46.2%) as compared to the ones undergoing ICSI in the presence of a clinical varicocele (31.4%) [73]. In their study, the chances of achieving a live birth (odds ratio = 1.87; 95% confidence interval 1.08–3.25; *P* = 0.03) by ICSI were significantly increased, while the chances of miscarriage occurrence after obtaining a pregnancy by ICSI were reduced (odds ratio = 0.433; 95% confidence interval 0.22 to 0.84; *P* = 0.01) had the varicocele been treated before assisted conception. 

Regarding the varicocele influence on androgen status, a recent report by Tanrikut et al. evaluated men with clinical varicocele in a case control study and demonstrated that testosterone levels were significantly lower compared to men without varicocele (416 × 469 ng/dL, *P* < 0.001) [74]. They also found that microsurgical repair was able to promote an improvement in testosterone levels of 178 ng/dL (mean) in over than 2/3 of patients.

## 7. Subclinical Varicocele: A Point of Debate

The definition of a subclinical varicocele is precisely what the term means: varicose veins from the pampiniform plexus which cannot be diagnosed solely by physical examination but rather depends on adjunctive diagnostic tools including Doppler examination, color Doppler ultrasound, scrotal thermography or venography [42, 43, 45–48, 75]. 

Currently, evidence to support recommendation to treat infertile men with subclinical varicocele is not convincing [38, 40, 49–51, 76, 77]. However, the benefit of bilateral varicocele repair in patients with a clinical varicocele at one side and a subclinical one at the contralateral side has been debated. In a comparative study involving 104 infertile men, no difference was observed in performing bilateral versus left only retroperitoneal surgical repair for left clinical and right subclinical varicocele [78]. Elbendary and Elbadry [79] reported a prospective series of 145 men with the same description. In their aforementioned study, varicocelectomy was performed using an inguinal open technique. Although a significant improvement in sperm parameters was observed in both groups, the magnitude of change in sperm count and motility and the spontaneous pregnancy rates were significantly higher in the group of men who had bilateral varicocele repair. Their findings are in agreement with early studies suggesting that bilateral varicocelectomy is more effective than unilateral for such patients [80, 81]. It is also hypothesized that there might be an alteration in blood flow following unilateral clinical varicocelectomy which may unmask an underlying contralateral venous anomaly resulting in a clinically manifested varicocele [81]. It should be stressed, however, that current guidelines do not support the repair of subclinical varicoceles [49–51].

## 8. Varicocele and Azoospermia: Is It Worth Treating?

Nonobstructive azoospermia (NOA) comprises a spectrum of altered testicular histopathology related with several diverse factors (genetic, gonadotoxins, trauma, infectious, etc.). Although infertile men presenting with NOA are the most difficult to treat, the recent advances in ART coupled with surgical methods of testicular sperm extraction (TESE) made it possible for approximately 20%–40% of men with NOA to father children of their own [82].

Varicocele is found in 5% of men with NOA, but its definitive role on the azoospermic status is still unknown [83]. The advent of ICSI renewed the interest about varicocele, as improvement in fertility potential may be decisive for infertile NOA men. A recent meta-analysis evaluated the impact of surgical repair in more than 200 men with clinical varicocele and NOA [70]. At a mean followup of 13 months, motile sperm was found in 39% of subjects; pregnancy was achieved in approximately 26% of men with sperm in the ejaculate, 60% unassisted, and 40% with IVF. Postoperative mean sperm density and motility were 1.6 million and 20%, respectively. Histopathology was the only predictor of success. Biopsy-proven hypospermatogenesis (HS) and maturation arrest (MA) were significantly more likely to correlate with finding sperm in the ejaculate than Sertoli-cell only (SCO) (odds ratio 9.4; CI 95% 3.2–27.3). The scarceness of randomized controls in the literature and the inclusion of case series without a control group in the aforementioned meta-analysis must be noted. Although an argument can be made that a control group would remain azoospermic, it is not rare to observe that NOA men occasionally ejaculate small quantities of motile sperm despite any intervention [84, 85]. 

Even after improvement of spermatogenesis in a subset of NOA men following varicocele repair, most individuals will remain azoospermic after varicocelectomy. Published data report 60% of success in sperm retrieval using testicular microdissection (micro-TESE) sperm extraction in NOA men who remain azoospermic after varicocele repair [86]. It is also suggested that varicocele repair may maximize the chances of retrieving sperm for ICSI in azoospermic men with clinical varicocele. Inci et al. reported a 2.6-fold increase in the chances of retrieving testicular sperm for ICSI using micro-TESE in NOA men with treated as compared to untreated varicocele [71].

## 9. New World Health Organization Reference Values for Human Semen Characteristics: Impact on the Recommendations for Treatment

In 2010, the World Health Organization (WHO) established new reference values for human semen characteristics, which are markedly lower than those previously reported [87]. Current guidelines propose that varicocele should be treated if palpable and in the presence of abnormal semen analyses [49–51]. 

Application of the new WHO reference values into clinical practice will result in patients previously deemed as candidates for varicocele repair now ineligible for treatment if their semen parameters are above the new cutoff reference values. At present, the number of men that fall into this category is unknown, as is the impact on their fertility potential of forgoing varicocele repair. Although no definitive conclusions can currently be drawn, the concern is that by denying these men a varicocele repair we may prevent them from achieving a substantial improvement in semen parameters and a greater chance of spontaneous pregnancy [88]. Men with a clinical varicocele and mild oligozoospermia or normozoospermia achieve higher spontaneous pregnancy rates after varicocelectomy than couples with moderate to severe oligozoospermia [89, 90]. Given the progressive deleterious effect of varicocele on testicular function [91, 92], one of the goals of treating varicocele is to halt the deterioration of sperm quality and prevent individuals with low “normal” semen parameters to cross into the defined infertile range. 

As such, the available data would support the practice of varicocelectomy for infertile men with clinical varicocele and low “normal” semen parameters according to the new WHO reference values. In addition, this knowledge challenges the current WHO recommendations for varicocele treatment and highlights the importance of a continuous debate.

## 10. Conclusions

Varicocele is a highly prevalent condition in the infertile male population. Its epidemiologic features suggest that it is a progressive pathology with genetic predisposition. Recent studies on the physiopathology of varicocele-related infertility have shown the likely influence of ultrastructural testicular changes and increased oxidative stress with implications on the seminal antioxidant capacity and sperm chromatin integrity. Controversy still remains regarding the benefit of varicocele repair to improve male fertility. Evidence exist both in favor and against it, but as of now, most specialty societies recognize that varicocele is detrimental to male reproductive health and its treatment may improve sperm function and chances of conceiving. The cornerstone of varicocele diagnosis remains the physical examination although ultrasound may be helpful in certain scenarios. Surgical treatment is the gold standard, and subinguinal microsurgical approach seems to offer the best results with fewer complications. Subclinical varicoceles should only be considered for treatment when associated with a contralateral clinical one. Fertility improvement in men with treated varicocele may have a favorable impact on assisted reproductive technology outcomes. In nonobstructed azoospermic men, varicocele repair may increase the likelihood of finding sperm in the ejaculates of men with biopsy-proven hypospermatogenesis or maturation arrest testicular histopathology or in the testis of those who remained azoospermic using sperm retrieval techniques. Lastly, the adoption of the newly released 2010 WHO reference values for semen parameters normality is likely to have a significant impact on varicocele treatment indication by excluding former candidates for varicocele repair based on the current recommendations for surgery. This should be looked at with caution so as not to miss the adequate timing to intervene and prevent testicular damage.
